# Accelerated phase contrast measurements of fetal blood flow using compressed sensing

**DOI:** 10.1186/1532-429X-18-S1-P30

**Published:** 2016-01-27

**Authors:** Christopher Roy, Mike Seed, Christopher Macgowan

**Affiliations:** 1 grid.17063.33Medical Biophysics, University of Toronto, Toronto, ON Canada; 2 grid.42327.300000000404739646Physiology and Experimental Medicine, The Hospital for Sick Children, Toronto, ON Canada; 3 grid.42327.300000000404739646Division of Pediatric Cardiology and Diagnostic Imaging, The Hospital for Sick Children, Toronto, ON Canada

## Background

Phase contrast (PC) MR is routinely used to quantify blood flow in postnatal subjects and through the use of metric optimized gating (MOG) has been employed in studies of fetal blood flow in both normal pregnancies and fetal congenital heart disease [1–3]. Still, the scan time required for high resolution fetal PCMR remains a practical limitation. Recently, compressed sensing (CS) has been integrated with MOG for accelerated CINE imaging of the fetal cardiac anatomy [4]. Here we examine the feasibility of CS for reconstructing retrospectively undersampled PC MR measurements of fetal vessels.

## Methods

Fully sampled PCMR data from the ascending and descending aorta were acquired in five fetal subjects (2 normal, 3 congenital heart disease). Typical fetal scan parameters where: VENC 150 cm/s, field of view 240 × 240 mm^2^, voxel size 1.25 × 1.25 × 5 mm^3^, TR/TE 6.6 ms/2.92 ms, 4 views per segment, scan time ~34 seconds. For each fully sampled data set MOG was performed to create time resolved CINE data sets which were then retrospectively undersampled (R = 2,4,6) and quantitatively compared to the fully sampled MOG data.

## Results

**Figure 1:**
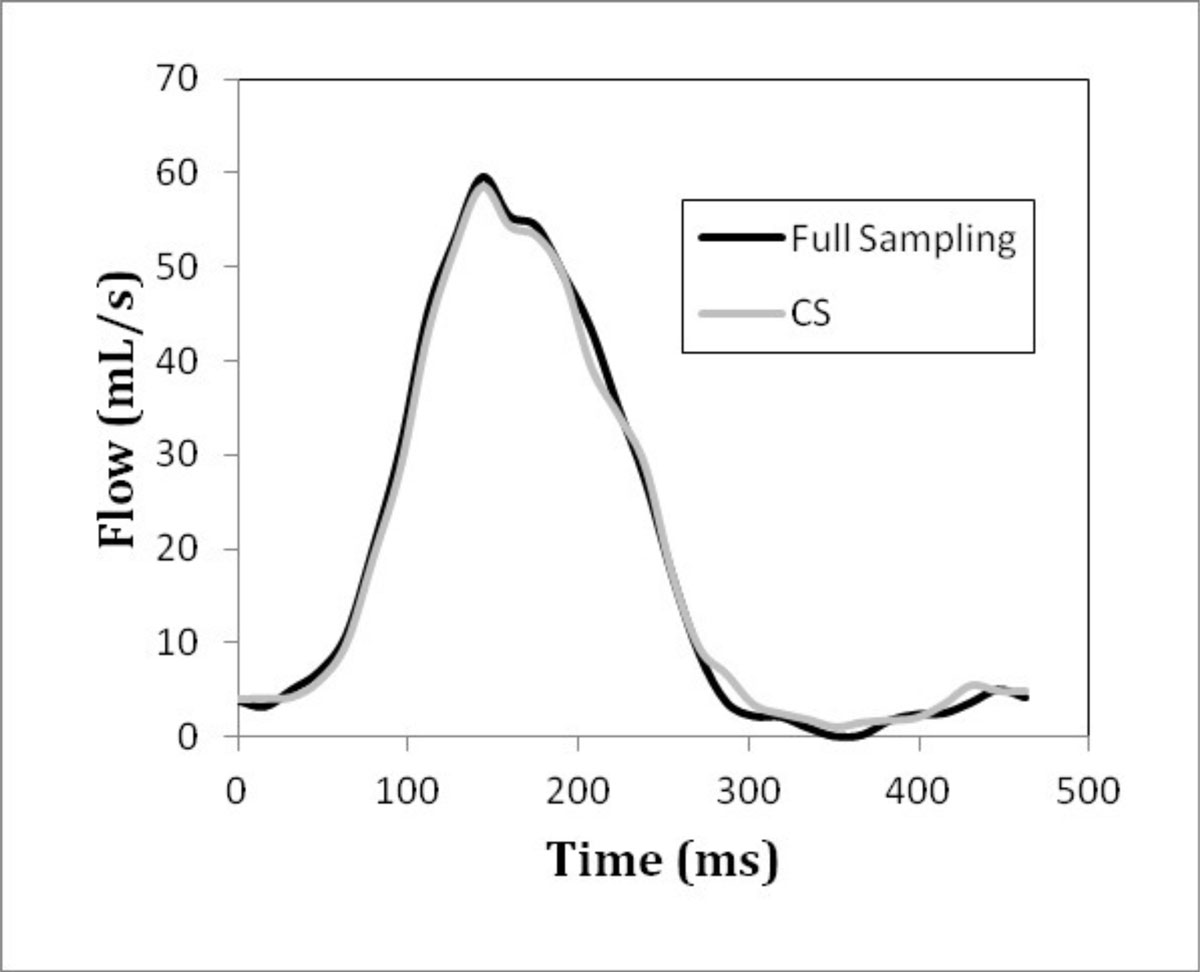
**Example flow curves through the healthy human fetal ascending aorta for fully sampled and undersampled (R=6) CS reconstructions**.

**Figure 2:**
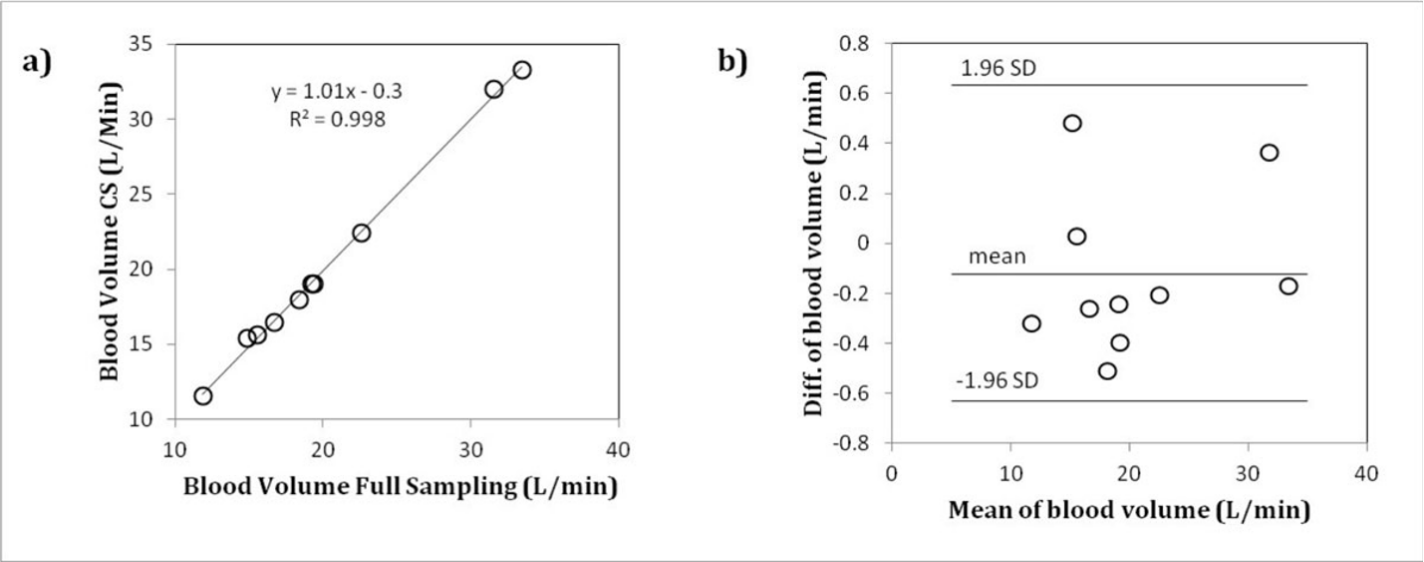
**Total blood volume per cardiac cycle for undersampled (R=6) versus fully sampled reconstructions**. a) Line plot. b) Bland-Altman plot.

Figure 1 shows an example flow curve of the human fetal ascending aorta for both fully sampled and undersampled (R=6) reconstructions. Figure 2 demonstrated comparable measurements of total blood volume per cardiac cycle between fully sampled and undersampled reconstructions (R=6). Finally a Bland-Altman plot (Figure 2b) shows good agreement between the two reconstruction methods.

## Conclusions

The feasibility of CS for reconstructing accelerated PC MR measurements of human fetal blood flow was accessed through retrospective undersampling of fully sampled MOG data. The results yielded accurate flow measurements for acceleration rates up to R=6. Further study using prospectively undersampled data is needed to evaluate this technique for clinical use.
